# Altered Functional Connectivity in Essential Tremor: A Resting-State fMRI Study

**DOI:** 10.1097/MD.0000000000001936

**Published:** 2015-12-11

**Authors:** Julián Benito-León, Elan D. Louis, Juan Pablo Romero, Juan Antonio Hernández-Tamames, Eva Manzanedo, Juan Álvarez-Linera, Félix Bermejo-Pareja, Ignacio Posada, Eduardo Rocon

**Affiliations:** From the Department of Neurology, University Hospital “12 de Octubre”, Madrid (JB-L, FB-P, IP); Centro de Investigación Biomédica en Red sobre Enfermedades Neurodegenerativas (CIBERNED) (JB-L, FB-P); Department of Medicine, Complutense University, Madrid, Spain (JB-L, FB-P, IP); Department of Neurology, Yale School of Medicine, Yale University, New Haven, CT, USA (EDL); Faculty of Biosanitary Sciences, Francisco de Vitoria University, Pozuelo de Alarcón (JPR); Neuroimaging Laboratory, Center for Biomedical Technology, Rey Juan Carlos University, Móstoles (JAH-T, EM); Department of Radiology, Hospital Ruber International, Madrid (JA-L); and Neural and Cognitive Engineering group, CAR, UPM-CSIC, CSIC, La Poveda – Arganda del Rey, Spain (ER).

## Abstract

Essential tremor (ET) has been associated with a spectrum of clinical features, with both motor and nonmotor elements, including cognitive deficits. We employed resting-state functional magnetic resonance imaging (fMRI) to assess whether brain networks that might be involved in the pathogenesis of nonmotor manifestations associated with ET are altered, and the relationship between abnormal connectivity and ET severity and neuropsychological function.

Resting-state fMRI data in 23 ET patients (12 women and 11 men) and 22 healthy controls (HC) (12 women and 10 men) were analyzed using independent component analysis, in combination with a “dual-regression” technique, to identify the group differences of resting-state networks (RSNs) (default mode network [DMN] and executive, frontoparietal, sensorimotor, cerebellar, auditory/language, and visual networks). All participants underwent a neuropsychological and neuroimaging session, where resting-state data were collected.

Relative to HC, ET patients showed increased connectivity in RSNs involved in cognitive processes (DMN and frontoparietal networks) and decreased connectivity in the cerebellum and visual networks. Changes in network integrity were associated not only with ET severity (DMN) and ET duration (DMN and left frontoparietal network), but also with cognitive ability. Moreover, in at least 3 networks (DMN and frontoparietal networks), increased connectivity was associated with worse performance on different cognitive domains (attention, executive function, visuospatial ability, verbal memory, visual memory, and language) and depressive symptoms. Further, in the visual network, decreased connectivity was associated with worse performance on visuospatial ability.

ET was associated with abnormal brain connectivity in major RSNs that might be involved in both motor and nonmotor symptoms. Our findings underscore the importance of examining RSNs in this population as a biomarker of disease.

## INTRODUCTION

Essential tremor (ET) is one of the most common adult neurological disorders,^1^ yet its pathogenesis is not well understood.^2–4^ The weight of emerging evidence is indicating that, aside from motor manifestations, ET is also associated with a number of nonmotor manifestations, including cognitive deficits,^5^ dementia,^6^ depressive symptoms,^7^ changes in sleep patterns,^8^ changes in visual reaction time,^9^ and hearing impairment.^10,11^ Clinical and imaging studies point to cerebellar involvement, and the tremor is thought to be mediated by a neuronal loop involving cerebello-thalamo-cortical pathways.^2–4^ In addition, tissue-based research has recently led to a newly proposed pathomechanistic model of ET – the cerebellar degenerative model.^12^

Among various advanced magnetic resonance imaging (MRI) techniques, functional MRI (fMRI) allows one to explore the dynamics of cortical functional reorganization.^13^ In most fMRI studies of ET conducted to date, an activation paradigm evoked by simple motor tasks or cognitive tasks was used.^13^ Task-related fMRI studies, however, have at least 1 major inherent limitation.^13^ This is the difficulty interpreting results due to large inter-subject variability in task performance, a problem that is even more pronounced among people who are trembling.^13^ This limitation of task-related fMRI studies is not a feature of a more recent approach – the acquisition of fMRI data during resting state conditions (ie, with subjects awake, but relaxed and not involved in any task).^14^ In this setting, spatially distributed networks of temporal synchronization can be detected that can characterize resting-state networks (RSNs).^14^ These RSNs have demonstrated high reproducibility across subjects, time, and research sites, and could serve as surrogate biomarkers for several neurological diseases, including Alzheimer disease, dementia with Lewy bodies, Parkinson disease, and multiple system atrophy, among others.^15,16^

To date, only 2 resting-state functional connectivity studies have been conducted with ET patients, and both have focused mainly on functional connectivity within the cerebello-thalamo-cortical network, which is related to motor symptoms.^17,18^ However, neither of these studies investigated RSNs integrity more broadly. Aside from motor symptoms, RSNs integrity could be involved in the pathogenesis of nonmotor manifestations associated with ET. These broader networks include the default mode network (DMN) and executive, frontoparietal, auditory/language, and visual networks. The 2 prior studies did not assess these RSNs, which might be important in the pathogenesis of other manifestations of ET.

The present study, using fMRI, compares resting-state functional connectivity in ET patients and healthy controls (HCs) and specifically assessed the following RSNs: DMN, executive control, 2 frontoparietal networks (left- and right-lateralized), as well as sensorimotor, cerebellar, auditory/language, and visual networks. Our a priori study hypotheses were as follows: several RSNs will be impaired in ET patients, including the DMN and the frontoparietal networks, since these RSNs are involved in cognition;^19,20^ and ET patients will show changes relative to HC in the cerebellum.

## METHODS

All procedures were approved by the ethical standards committees on human experimentation at the University Hospital “12 de Octubre” (Madrid). Written (signed) informed consent was obtained from all enrollees.

### Participants

ET patients were consecutively recruited from October 2012 to July 2013 from the outpatient neurology clinics of the University Hospital “12 de Octubre” in Madrid (Spain). Two neurologists with expertise in movement disorders (JB-L and JPR), who were blinded to the MRI results, examined the patients and used the Fahn–Tolosa–Marìn tremor rating scale to assign a total tremor score (range = 0–144).^21^

Diagnoses of ET were assigned by the 2 neurologists (JB-L and JPR) using the Consensus Statement on Tremor by the Movement Disorder Society.^22^ Furthermore, all ET patients had a normal [(123) I]FP-CIT single photon emission computed tomography scan. Patients with history of stroke, epilepsy, or head injury were excluded. Furthermore, based on a detailed clinical mental status examination, we excluded patients with Diagnostic and Statistical Manual of Mental Disorders-IV criteria for dementia.^23^ All ET patients underwent a detailed videotaped neurological examination. Each videotape was reviewed by a senior neurologist specializing in movement disorders (EDL) who re-assessed ET diagnosis using the Consensus Statement on Tremor by the Movement Disorder Society.^22^ The ET patients were also followed at regular intervals (3, 6, or 12 months, based on clinical need) after the MRI procedure, and their clinical assessment, described above, was repeated. The mean duration of follow-up after the MRI procedure was 2.5 years (median = 2.7 years; range = 1.8–2.8 years).

HCs were recruited either from relatives or friends of the health professionals working at the University Hospital “12 de Octubre” of Madrid (Spain) or among the relatives of patients who came to the neurological clinics for reasons other than ET (eg, headache, dizziness). None reported having a first-degree or second-degree relative with ET. Each control was examined by two neurologists (JB-L and JPR), who were blinded to the MRI results, to further rule out any neurological conditions.

### Neuropsychological Testing

All participants underwent a detailed neuropsychological assessment covering the domains of attention, executive function, verbal memory, visual memory, visuospatial ability, and language. Testing was performed by a trained neuropsychologist (VP, see acknowledgments) using standardized procedures. These tests have previously been described.^24^ Raw scores were transformed into z scores based on the mean and standard deviation (SD) values from HC. Higher z scores indicated better performance. Depressive symptoms severity was measured by the original 17-item version of the Hamilton depression rating scale.^25^

The tasks from the neuropsychological and depressive symptoms assessment were z-standardized, averaged, and compiled to create 6 composites scores (attention, executive function, verbal memory, visual memory, visuospatial ability, and language) for each participant. Each composite score was then employed as a continuous variable in subsequent regression analyses.

### MRI Procedure

Patients and controls were positioned in the scanner and were told to relax with their eyes closed. They were immobilized with a custom-fit blue bag vacuum mold (Medical Intelligence, Inc., Germany.) to prevent motion artifacts. Earplugs and noise-reduction headphones were used to attenuate scanner noise. During the data acquisition for functional connectivity, the participants were instructed not to engage in cognitive or motor activities. The functional run required 6 minutes to complete.

Images were acquired on a General Electric Signa 3T MRI Scanner (General Electric Healthcare, Fairfield, CT) using a whole-body radiofrequency coil for signal excitation and quadrature 8-channel coil for reception. Resting-state fMRI data consisted of 120 volumes of a repeated gradient-echo echo planar imaging T2∗-weighted sequence whose parameters were repetition time (TR) = 3 s, echo time (TE) = 28 ms, voxel dimensions = 2.7 × 2.7 × 2.8 mm, 39 oblique ACPC-oriented slices, flip angle = 90°, and 6 dummy scans.

For the structural image, a high-resolution, 3-dimensional T1-weighted gradient Echo-SPGR was acquired with the following parameters: TR = 9.2 ms, TE = 4.128 ms, inversion time (TI) = 500 ms, field of view = 240 mm, acquisition matrix = 240 × 240, slice thickness = 1 mm, full brain coverage, resolution = 1 × 1 × 1 mm, flip angle = 120°, and 166 sagittal slices.

### Image Preprocessing

Resting-state fMRI images were analyzed using FSL (fMRIB Software Library; available at: www.fmrib.ox.ac.uk/fsl) and AFNI (Analysis of Functional NeuroImages; available at: http://afni.nimh.nih.gov/afni/).^26,27^ The preprocessing included the following steps: de-spiking, slice timing correction, motion correction, field map correction, spatial smoothing (full-width half maximum = 6 mm), temporal high pass filtering (cut-off of 100 s), functional to anatomical image registration, and normalization to the atlas space of the Montreal Neurological Institute (MNI) 152 T1 2 mm template. Despiking was done using AFNI and the rest of the steps of the preprocessing pipeline were done with FSL.

### Image Analysis

Resting-state fMRI data were analyzed using independent component analysis (ICA), in combination with a “dual-regression” technique.^28,29^ This method automatically determines the most consistent RSNs, based on an assessment of the similarity of predefined templates.^28,29^

In order to obtain the group independent spatial maps identifying RSNs across all participants, the multivariate exploratory linear optimized decomposition into independent components toolbox in FSL (fMRIB Software Library; available at: www.fmrib.ox.ac.uk/fsl) was used. A Temporal Concatenation Group ICA restricting the number of components to 25 was performed to study large-scale spatial networks.^30^ Data from all subjects, patients and controls, were concatenated for this analysis.

The 25 independent components (IC) were sorted into 2 broad classes: biologically plausible/functionally relevant components or RSNs, and scanner/physiological artifactual components (cerebrospinal fluid, white matter, head motion, and large vessels artifacts). The inspection was made visually based, on each component's spatial profile and time course following criteria purposed by Kelly et al.^31^ Eight RSNs previously related to functionally relevant brain functions^32^ were identified: DMN, executive network, 2 frontoparietal networks (left- and right-lateralized), and sensorimotor, cerebellar, auditory/language, and visual networks.

These 8 ICs spatial maps were used as the RSN spatial map templates in the first step of the subsequent dual regression analysis.

The image analysis was performed in 2 steps with FSL-dual regression:^33^ each RSN spatial map template was used as a mask in a spatial regression against each individual fMRI dataset in order to obtain a subject-specific time course associated with that RSN. The individual time courses related to each RSN spatial map template in the first regression were then used in a temporal regression to estimate a subject-specific spatial correlation map for each RSN. After this dual regression, spatial maps of all subjects were collected for each original RSN.

Permutation statistics were computed with FSL-randomize to evaluate functional connectivity differences between the 2 groups in each RSN using the previously obtained subject-specific spatial maps (number of permutations = 1000). We statistically accounted for the effects of age and sex by including these variables as covariates in the statistical model. The dual regression considered the whole brain, not only the areas where each RSN was strongly manifested. Results were considered significant for *P* < 0.005 uncorrected using a threshold-free cluster enhancement.^34^ The following information was provided for the clusters whose size was greater than or equal to 10 voxels (80 mm^3^): maximum threshold-free cluster enhancement *P* value uncorrected of the cluster (permutation statistics), cluster size, MNI coordinates of the maximum of the cluster, Talairach atlas label of this region, and the corresponding Brodmann area or the most probable lobule reported in the cerebellar atlas in MNI152 space, after normalization with FLIRT.

### Sample Size and Statistical Analyses of Clinical and Neuropsychological Data

In several recent publications on suitable sample sizes for functional neuroimaging studies, it has been found that a group size of approximately 20 is sufficient.^35,36^

Statistical analyses for the clinical and neuropsychological measures were conducted using SPSS 21 (Statistical Package for the Social Sciences). Mean scores (age and neuropsychological variables) were compared using 2 independent sample *t*-tests for continuous and normally distributed data, and Mann–Whitney *U* test for nonnormally distributed data, where appropriate. The χ^2^ test was used to analyze differences in sex distribution.

The mean z scores of the clusters from the RSNs that were significant after group comparison were regressed against age, sex, educational level, severity of ET, disease duration, and each 1 of the 6 different cognitive composite measures, and the 17-item Hamilton depression rating scale score. A value of *P* < 0.05 was considered statistically significant.

## RESULTS

### Clinical and Neuropsychological Testing Results

Of the 26 ET patients who were initially eligible, 2 had ET with dystonic features after reviewing their videotapes and were therefore excluded. One was excluded from the final analyses because he developed incident Parkinson disease during that follow-up. None of the patients and controls were excluded because of neurological comorbidities or structural abnormalities on conventional MRI images. A strict criterion for head movements assessment was adopted (maximal absolute head movement less than 1.0 mm and 1.0° in the x, y, and z directions). That is why 1 HC was excluded from the analysis due to this criterion.

The final sample included 23 right-handed ET patients (12 women and 11 men) and 22 right-handed HC (12 women and 10 men). The 23 ET patients did not differ to a significant degree from the 22 controls in terms of age, gender, and educational level (Table 1). The mean tremor duration was 22.9 ± 16.5 years and the mean total severity tremor score was 29.3 ± 15.7 (Table 1).

**TABLE 1:**
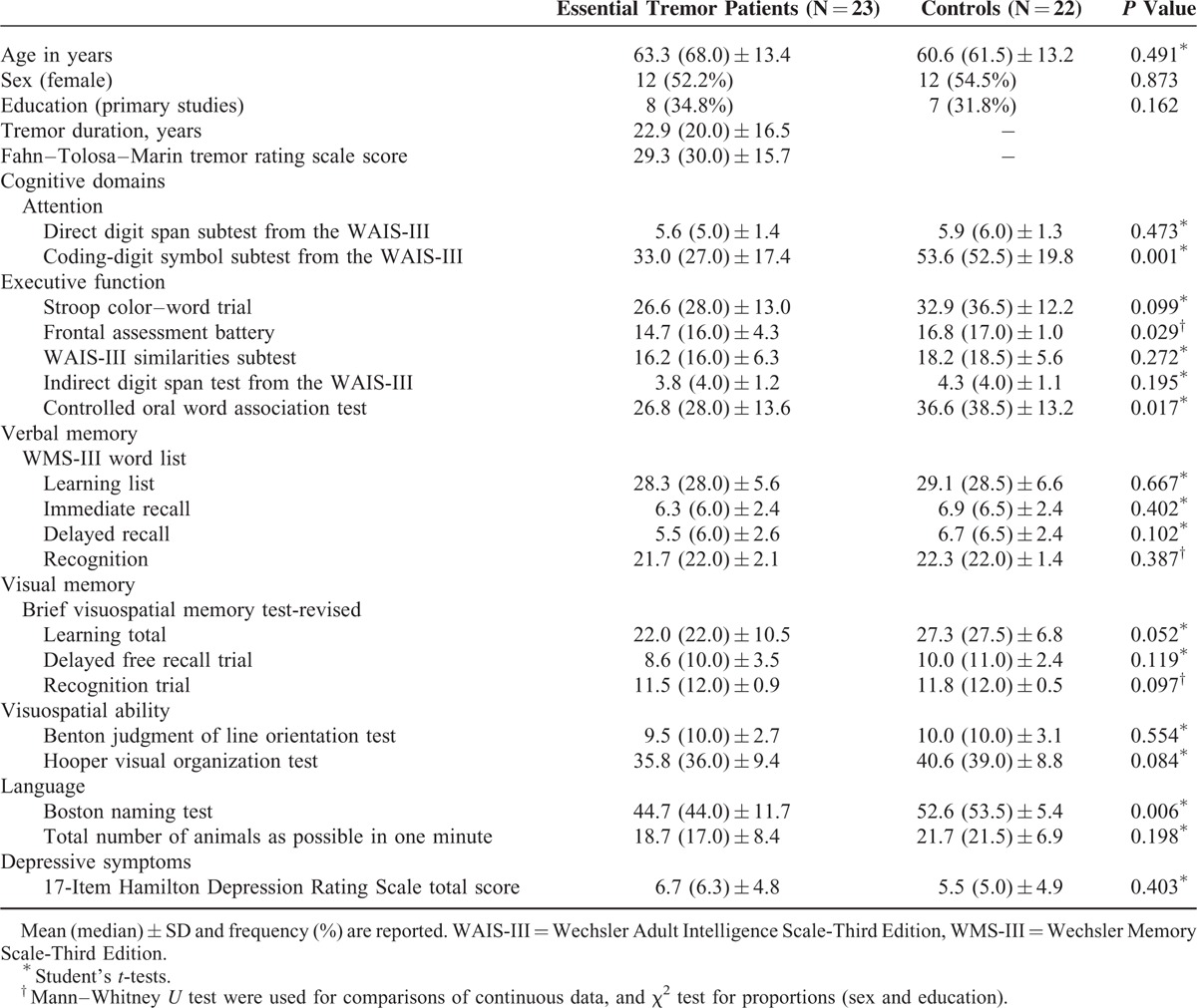
Comparison of Demographic, Clinical and Cognitive Domains of Essential Tremor Patients Versus Healthy Controls

The results of neuropsychological testing are shown in Table 1. In several domains, ET patients’ cognitive performance was significantly worse than that of the HC. These differences involved selected tests of attention, executive function, and language.

### Resting-State fMRI Results

**FIGURE 1:**
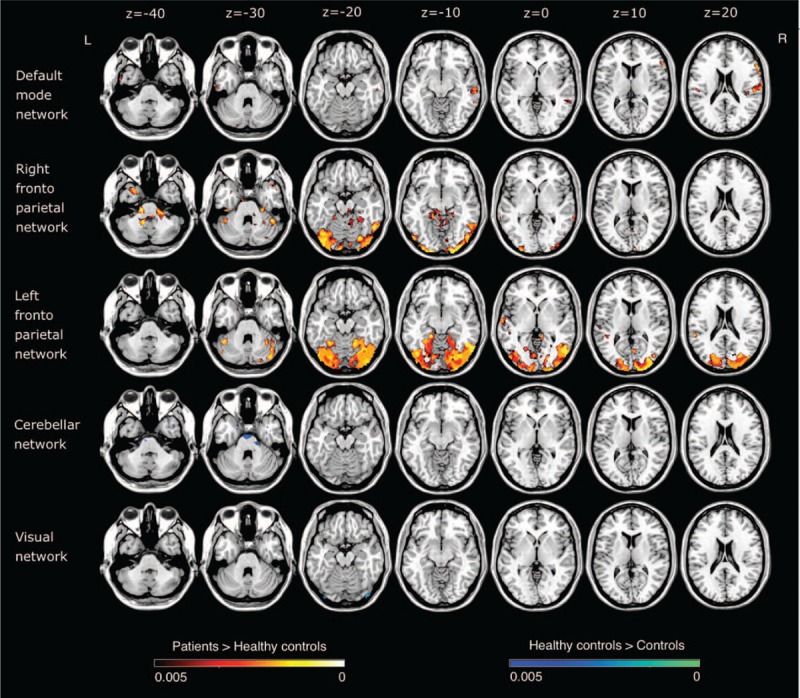
Resting state brain networks showing significant functional connectivity differences between the essential tremor (ET) patients and healthy control group.

All results for the RSNs, which showed between-group functional connectivity differences, including MNI coordinates and *P*-values for peak voxels of all statistically significant clusters, are summarized in Table 2 and visualized in Figure 1.

**TABLE 2:**
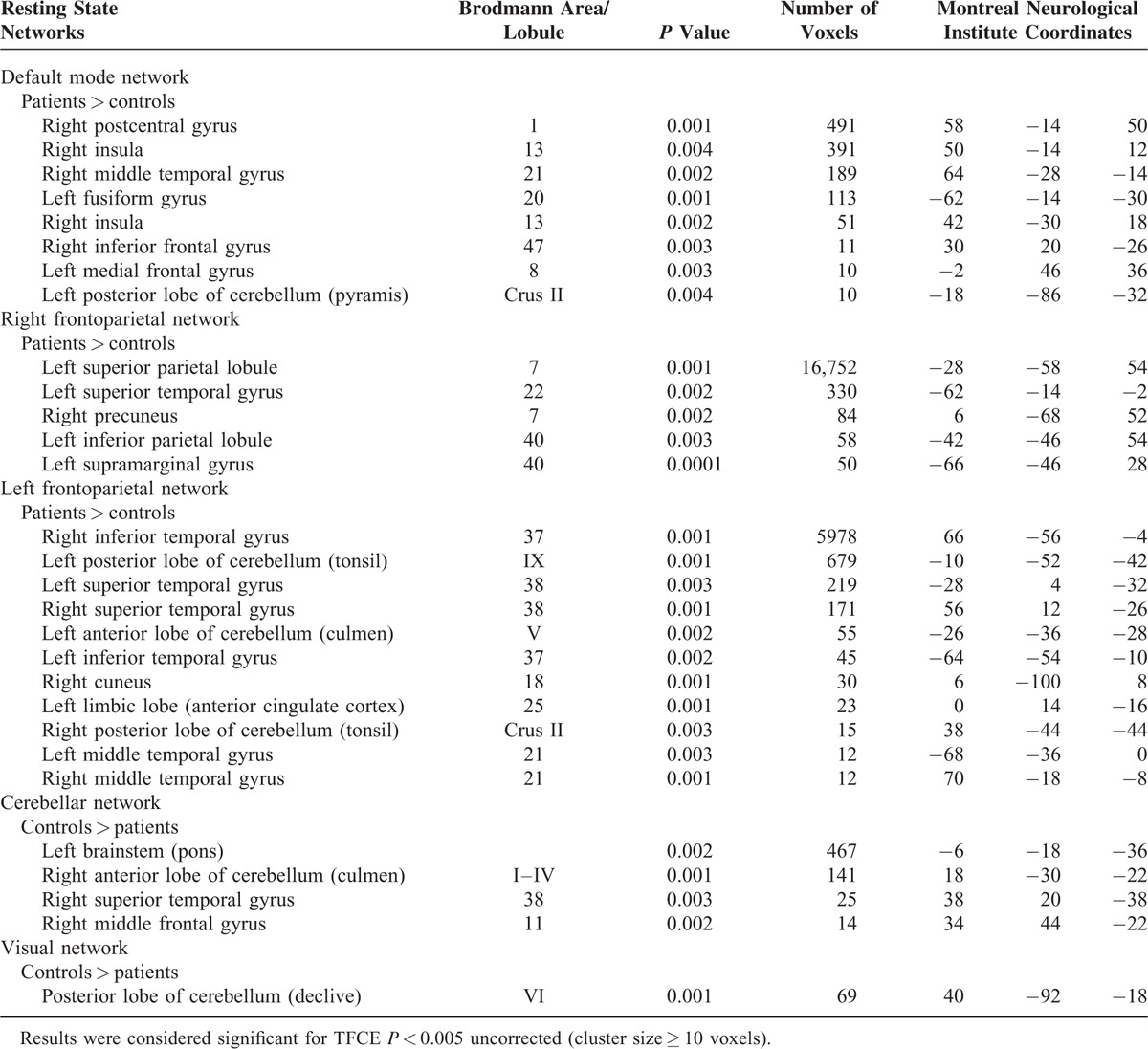
Regions That Showed Statistically Significant Differences in Functional Connectivity

#### DMN

The DMN involves the precuneus, posterior cingulate cortex, ventro-medial prefrontal cortex, lateral parietal cortices, and bilateral hippocampi.^32^ The between group comparison revealed significantly greater connectivity in ET patients compared to HC in areas involving the right postcentral and middle temporal gyri, right inferior and left medial frontal gyri, left fusiform gyrus, the right insula as well as the left posterior lobe of cerebellum.

#### Executive Network

The executive network, which includes the anterior cingulate and paracingulate cortex, bilateral inferior and superior frontal gyri, and bilateral angular gyri, is involved in executive functions, such as control processes and working memory.^32^ This network was virtually identical for ET patients and HC.

#### Frontoparietal Networks

The frontoparietal network is represented by 2 distinct but specular components in the right and left hemisphere.^32^ The frontoparietal network involves the inferior frontal gyrus, the medial frontal gyrus, the precuneus, the inferior parietal, and the angular gyrus.^32^ This RSN has been associated with different functions such as memory, language, attention, and visual processing.^32^

In the right frontoparietal RSN, there was increased connectivity in ET patients in the left superior temporal, inferior parietal lobule, and supramarginal gyri, as well as in the right precuneus. Meanwhile, for the left frontoparietal RSN, both the cerebellum (right posterior lobe and left posterior and anterior lobes) and the temporal lobes showed increased connectivity in ET patients. Additionally, the left limbic lobe and the right cuneus showed increased connectivity in HC.

#### Cerebellar Network

The cerebellar network consists of the cerebellum and areas of the thalamus.^32^ The comparison between the 2 groups showed significantly lesser connectivity in ET patients in the right superior temporal gyrus, right middle frontal gyrus, left pons, and on right anterior lobe of cerebellum.

#### Visual Network

The visual network involves the medial, occipital pole, and lateral visual areas.^32^ The lateral visual network encompasses higher-order visual processing areas including bilateral lateral occipital cortices, bilateral occipital fusiform gyri, and parts of the occipitotemporal junction.^32^ These areas are associated with motion processing, visual attention, and cognition-space paradigms involving visual integration from retinotopic visual fields to egocentric frames useful in executive processes.^32^ On the other hand, the medial visual network subsumes many early visual processing areas including the occipital pole, cuneus, and areas of the precuneus.^32^ These areas comprise V1 and V2 visual cortex involved in early line orientation and color distinction.^32^ With respect to visual network, HCs were found to have increased connectivity compared to ET in posterior lobe of cerebellum.

#### Sensorimotor Network

The sensorimotor network includes the precentral gyrus, the postcentral gyrus, and the supplementary motor area, which are all normally involved in motor tasks.^32^ This network was virtually identical for ET patients and HC.

#### Auditory/Language Network

The language/auditory network includes superior temporal gyrus, Heschl gyrus, and posterior insula, corresponding most strongly with speech execution, language paradigms, and auditory perception.^32^ Again, the network was virtually identical for patients and HC and, thus, no significant difference was obtained between both groups.

### Relationships Between Functional Connectivity, Disease Variables (Severity and Duration of Disease), and Cognition

These correlations were calculated in ET patients only, and only for the voxels that showed differences between patients versus HC (see Table 2). There was an association between disease severity and connectivity in the DMN, and between disease duration and connectivity in the DMN and the left frontoparietal network (Table 3). In addition, increased connectivity in 3 RSNs (DMN and frontoparietal networks) was associated with cognitive processes (attention, executive function, visuospatial ability, verbal memory, visual memory, and language) and depressive symptoms (Table 3). Further, in the visual network, decreased connectivity was associated with worse performance on visuospatial ability (Table 3).

**TABLE 3:**
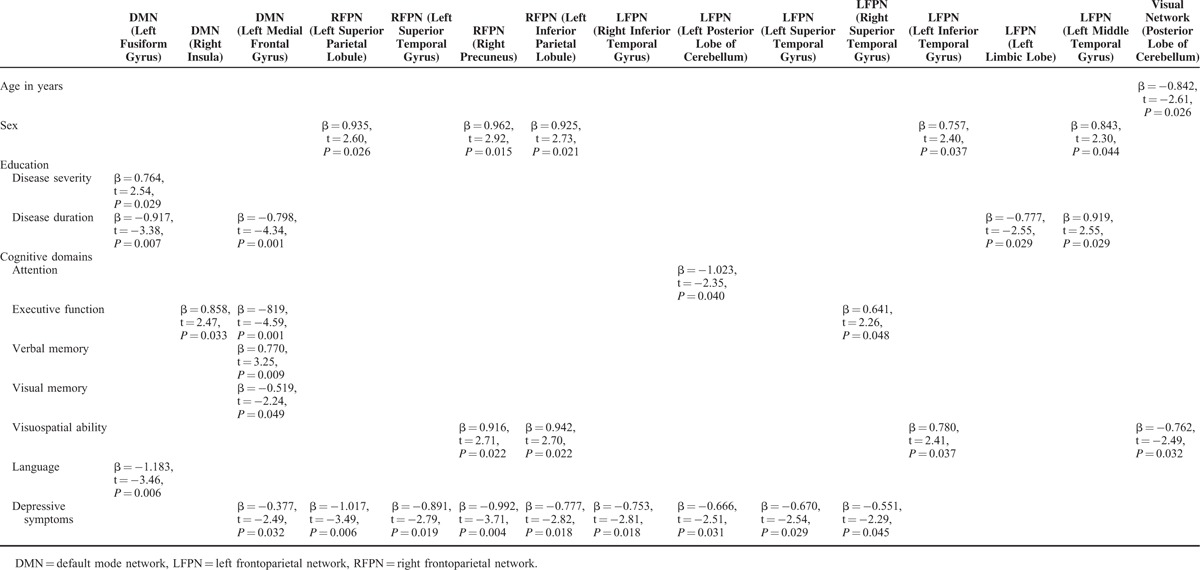
Associations of Demographic, Disease, and Cognitive Variables and the Mean z Value of the Significantly Differing Voxels of Functional Connectivity in all Essential Tremor Patients

## DISCUSSION

In the current study, we investigated functional connectivity in RSNs that were hypothesized to be relevant to both motor and nonmotor symptomatology in a sample of ET patients and controls. Overall, relative to HC, ET patients showed increased connectivity in RSNs involved in cognitive processes (DMN, as well as in frontoparietal networks), and decreased connectivity in the cerebellum and visual networks. Changes in network integrity were associated not only with ET severity (DMN) and ET duration (DMN and left frontoparietal network), but also with cognitive ability. Specifically, in at least 3 networks (DMN and frontoparietal networks), increased connectivity was associated with worse performance on different cognitive domains and depressive symptoms. In addition, in the visual network, decreased connectivity was associated with worse performance on visuospatial ability.

At first glance, the presence of increased connectivity seems counterintuitive; however, this is also found in mild cognitive impairment, early multiple sclerosis, and diabetes.^37–39^ In general, RSNs are functionally connected, and dysfunction in 1 network may lead to dysfunction in the other networks.^40^ Reduced functional connectivity is thought to reflect dysfunction of the network, and increased functional connectivity has been interpreted as a compensatory mechanism or reorganization of the network.^40^

There are 2 previous resting-state fMRI studies investigating brain functional connectivity in ET patients, although they differed substantially from ours. Both studies focused mainly on functional connectivity within the cerebello-thalamo-cortical network, which is related to motor symptoms. Neither of these studies investigated RSN integrity more broadly. Aside from motor symptoms, RSN integrity could be involved in the pathogenesis of nonmotor manifestations associated with ET. Popa et al,^17^ studying 11 ET patients and 11 HC at baseline, used ICA to quantify resting-state fMRI connectivity between the cerebellum and the motor network, and between the cerebellum and the DMN. The study was repeated in patients after 5 days of bilateral 1 Hz repetitive transcranial magnetic stimulation over the cerebellum.^17^ Before repetitive transcranial magnetic stimulation intervention, both the motor cerebellum and the cerebellar vermis showed impaired functional connectivity with the rest of the cerebello-thalamo-cortical and DMN, respectively.^17^ Using regional homogeneity (ReHo) of resting-state fMRI as a metric, Fang et al^18^ reported local functional connectivity abnormalities in 20 ET patients compared with 20 HC, mainly located in the cerebello-thalamo-cortical motor pathway and nonmotor cortices (including prefrontal, parietal, and insular lobes). The authors speculated that the abnormal ReHo in those nonmotor cortices may be related to nonmotor symptoms (eg, cognitive, psychiatric, and sensory deficits) seen in ET.^18^ However, that assertion is rather difficult to make, since changes in those nonmotor cortical areas were not correlated with nonmotor clinical data, such as neuropsychological profile data of the participants, which was not measured by the authors.^18^ In addition, the ReHo method is fundamentally local in nature and therefore exhibits a high degree of sensitivity to different levels of spatial smoothing.^41^ Further, the insensitivity of the ReHo method to shape differences between clusters precludes drawing inferences on the degree of correspondence between spatially remote regions, making it difficult to characterize the RSNs.^41^

Although the sensorimotor and the visual and auditory networks involve cortical regions normally engaged in sensorimotor, visual, and auditory processes, respectively, the DMN and the executive and the frontoparietal networks are the RSNs most relevant for cognition.^19,20^ We found increased functional connectivity in the DMN and both frontoparietal networks in patients with ET. It has been reported that ET patients have some changes in cognitive functions, which rely on the DMN, executive, and frontoparietal networks.^19,20,24^ Of additional interest, we found the right insula to be more strongly connected to the DMN in patients with ET. Several neuroimaging data reveal that the insular cortex is involved in various neuropsychiatric diseases.^42,43^ One may speculate that these insular changes in ET might be an early marker of nonmotor manifestations that have been related to ET, such as cognitive impairment, dementia, and mood disorders. However, this possibility requires further study.

Our results also indicate that ET patients present a certain decreased functional connectivity in visual network. The aberrant functional connectivity of visual network found in our study could be associated with perceptual impairments in ET patients; however, clinical studies have yet to study or document such changes. By contrast, despite the existing body of research on abnormal hearing in ET,^10,11^ there were no group differences in functional connectivity in the auditory network.

The study was not without limitations. First, it would be of interest to perform a comparative study with other diseases characterized by tremor (eg, Parkinson disease). Second, the diagnosis of ET was based on clinical criteria and further supported by normal [(123) I]FP-CIT single photon emission computed tomography scan results. None of the ET cases had post-mortem assessments, so that it was not possible to determine whether they had the types of changes that have been reported in ET.^44^

In closing, we observed selective impairments of RSNs intrinsic functional connectivity in ET patients. This and other resting-state fMRI studies might provide a novel method to understand the pathophysiological mechanism of motor and nonmotor features of ET. Further, our findings suggest that resting-state fMRI may prove useful as a biomarker in ET.

## Acknowledgments

The authors thank neuropsychologist Verónica Puertas, for her assistance with the project.
